# Amorphous Solid Dispersion of Epigallocatechin Gallate for Enhanced Physical Stability and Controlled Release

**DOI:** 10.3390/ph10040088

**Published:** 2017-11-09

**Authors:** Yizheng Cao, Jing Teng, Jon Selbo

**Affiliations:** Solid State Chemical Information (SSCI), a Division of Albany Molecular Research Inc., West Lafayette, IN 47906, USA; yizheng.cao@amriglobal.com (Y.C.); jon.selbo@amriglobal.com (J.S.)

**Keywords:** epigallocatechin gallate, amorphous solid dispersion, physical stability, controlled release, bioavailability

## Abstract

Epigallocatechin gallate (EGCG) has been recognized as the most prominent green tea extract due to its healthy influences. The high instability and low bioavailability, however, strongly limit its utilization in food and drug industries. This work, for the first time, develops amorphous solid dispersion of EGCG to enhance its bioavailability and physical stability. Four commonly used polymeric excipients are found to be compatible with EGCG in water-dioxane mixtures via a stepwise mixing method aided by vigorous mechanical interference. The dispersions are successfully generated by lyophilization. The physical stability of the dispersions is significantly improved compared to pure amorphous EGCG in stress condition (elevated temperature and relative humidity) and simulated gastrointestinal tract environment. From the drug release tests, one of the dispersions, EGCG-Soluplus^®^ 50:50 (*w*/*w*) shows a dissolution profile that only 50% EGCG is released in the first 20 min, and the remains are slowly released in 24 h. This sustained release profile may open up new possibilities to increase EGCG bioavailability via extending its elimination time in plasma.

## 1. Introduction

**Figure 1:**
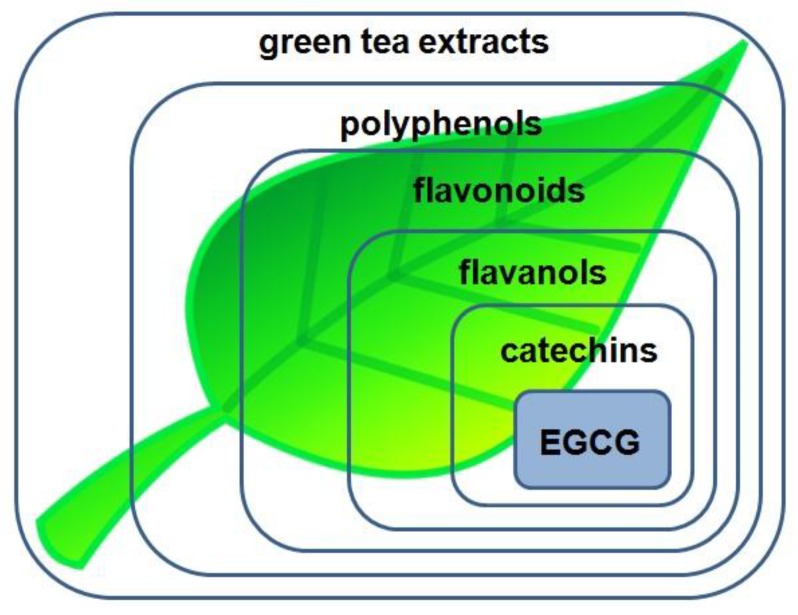
Hierarchical terminology of green tea extracts down to epigallocatechin gallate (EGCG).

Green tea has been one of the most popular and consumed beverages, widely recognized for its healthy influences, since ancient times [1]. Lately, the benefits of green tea, such as its antiarthritic, antibacterial, antiangiogenic, antioxidative effects [2], have been extensively reported. The benefits are mainly attributed to the polyphenols, of which catechins is the major component, including epicatechin (EC), epigallo-catechin (EGC), epicatechin-3-gallate (ECG), and epigallocatechin gallate (EGCG). Catechins are widely considered to be preventive agents against mammary cancer post-initiation, degenerative diseases, oxidative stress, cardiovascular and neurological disorders, and hepatotoxicity [2]. They are also antitumorigenic agents and immune modulators in immunodysfunction caused by transplanted tumors or cancer treatments [2]. Many of these health beneficial effects are credited to the most abundant catechin: EGCG [3,4]. While a plethora of sources have joined the advocacy for the magic of tea extracts, the terminology is sometimes misleading with respect to the classification and scope of the different ingredients. Figure 1 summarizes the commonly used hierarchical nomenclature from the broadly named “green tea extracts” down to EGCG.

**Figure 2:**
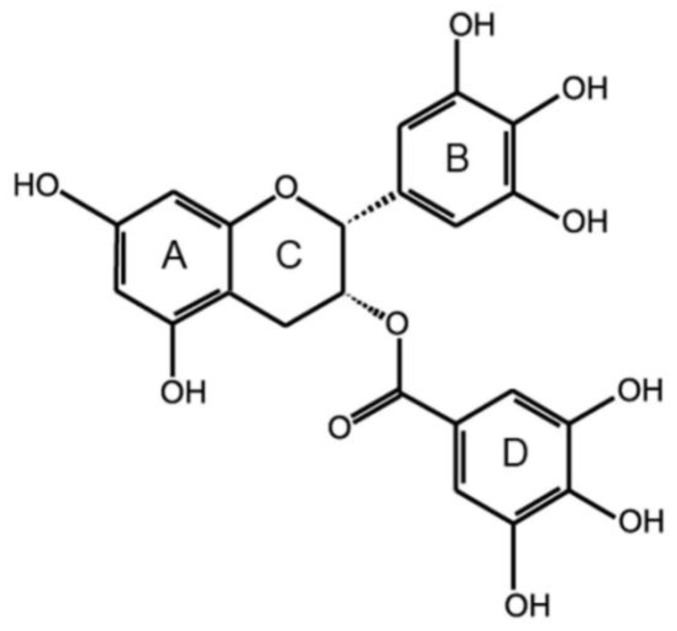
Chemical structure of EGCG.

The chemical structure of EGCG is provided in Figure 2. As the most abundant catechin (accounting for 60–70% of total tea catechins [5]), it is usually used as a quality indicator [6] and is claimed to be the most prominent catechin regarding the beneficial influence to health [7]. It is also one of the most extensively explored polyphenolic components [8,9] due to the strong antioxidant and cancer chemopreventive properties [10,11], as well as the only polyphenol presence in plasma at a high proportion (77–90%) in free form [12]. To date, EGCG has been demonstrated to be an anticancer [13,14,15], antioxidant, and antibacterial [16] agent, and also chemopreventive, anti-inflammatory, and anti-aging in topical treatments [17]. It is also reported to be protective against cardiovascular [18,19,20] and neurodegenerative diseases [21,22], UV-induced photodamage, basal cell carcinomas, melanomas, skin papillomas [23,24], obesity, and diabetes [2]. In addition, EGCG has shown interactions with a number of proteins such as α-synuclein, amyloid-β, and huntingtin, with a redirection to non-toxic species or remodeling of fibrils [25].

While the vast potential of EGCG in health care has been claimed, two major issues are identified that affect its utilization: high instability and low bioavailability. The instability of green tea catechins has been under study for several decades [6]. EGCG has shown considerable instability and degradability at solid state and in solutions [26,27]. A cow study found that catechins including EGCG are substantially degraded by rumen microorganisms resulting in no detectable catechins in plasma; however intraduodenal administration improved plasma concentration of all catechins with increasing dosage [28,29,30]. The instability of EGCG has crucially affected its processing, storage [6], and as expected, dosing in the gastrointestinal (GI) tract. In the meantime, the poor bioavailability of EGCG has been reported in a number of articles for rodents [31] and humans [32], with values down to less than 2–5% [33,34]. Although mechanisms responsible for the poor bioavailability have not been fully understood, the instability/degradation of EGCG in the GI tract, and its rapid in vivo dissolution and elimination [12,35,36] are believed to be important causes. The reported elimination half-life of EGCG is 3.4 ± 0.3 h [37].

A large amount of endeavors worldwide have been undertaken to unleash the prospects of EGCG. Lambert et al. [38] reported an improvement of 30% in bioavailability with co-administration of piperine, an alkaloid from black pepper, as a result of the extended GI transit allowing for longer residence time in the intestine. However it is concerned that consumption of piperine may influence the metabolism of food and drugs, bringing potential negative effects [39]. A more commonly hypothesized approach to improve the bioavailability is to decrease the dissolution rate and solubility, and therefore establish a sustained release of EGCG in the GI tract. Methods such as encapsulation or making insoluble complex [33,40] result in a slower release of EGCG from the capsulated structure/complex, which also diminishes its chemical degradation in the GI tract. Shutava et al. [16] manifested a layer-by-layer assembly to encapsulate EGCG with gelatin. Patel et al. [33] fabricated colloidal EGCG-methylcellulose complexes in aqueous suspensions resulting in a sustained release spanning two hours in both simulated intestinal and gastric fluids. However the release data after the first two hours were not provided; and preparation procedures of such colloidal complex seemed difficult to control. Smith et al. doubled the oral bioavailability with a nanolipidic formulation [39], which however involved an alcohol suspension, and therefore limited the application. In a later work, the same group attempted to generate different forms of EGCG cocrystals to lower aqueous solubility [30], whereas it was found that merely decreasing solubility by up to one order of magnitude only slightly enhanced the bioavailability.

In this work, the approach of amorphous solid dispersion is employed targeting the enhancement of the bioavailability and physical stability of EGCG. Amorphous materials have been substantially used in the pharmaceutical industry due to the high solubility. However pure amorphous API (active pharmaceutical ingredient) is rarely marketed because of the physical instability, i.e., the tendency towards crystallization during storage and processing. Amorphous solid dispersion is a paradigm that kinetically stabilizes amorphous API via the presence of excipients, typically polymers, to help prevent crystallization and maintain supersaturation [41]. Owning to the substantial length and flexibility of the polymer chains, the API is separated into interstitial solid solution with the molecules in the interstices. Via this confinement and immobilization, the amorphous API is significantly stabilized compared to its neat form. For a thorough review on amorphous solid dispersion, readers are directed to the article by Baghel et al. [42].

This work employs a convenient lyophilization approach to generate EGCG amorphous solid dispersions targeting two objectives: (1) improve physical stability of EGCG in common storage/processing and simulated GI environments; (2) establish a sustained release that may potentially enhance oral bioavailability. To the best knowledge of the authors, this is the first work generating EGCG amorphous solid dispersions aiming at minimize these two critical issues.

## 2. Results

### 2.1. Initial Screen

Nine polymers that are commonly used as pharmaceutical excipients were initially screened for their solubility (in water and dioxane), compatibility with EGCG in solvents, and crystallinity after lyophilization (for amorphous solid dispersion preparation purpose). For each polymer, individual solutions of polymer (in dioxane) and EGCG (in water) were prepared and then slowly mixed together as described in Section 3.1. The initial screen results are provided in Table 1. Upon mixing, precipitates were observed in the mixtures containing EGCG with polyvinylpyrrolidone (PVP K-90), polyvinyl alcohol (PVA), polyethylene glycol (PEG), and polyvinyl acetate (PVAc), and therefore these samples did not move forward with dispersion preparation. The other five polymers, i.e., hydroxypropyl methylcellulose acetate succinate (HPMCAS), hydroxypropyl methylcellulose phthalate (HPMCP), Soluplus^®^, cellulose acetate, Gelucire^®^ 50/13 appeared to be compatible with EGCG.

Precipitates observed upon mixing EGCG with PVP, PVA, PEG, and PVAc are due to binding between EGCG and polymers resulting in colloids and aggregates [33]. These colloids may find applications of special formulations in food or drugs, whereas they could also raise difficulties in formulation and reduce efficacy of polyphenols [33]. Furthermore, weak binding also occurred between EGCG and the other five polymers, since during mixing of solutions, samples can be cloudy but clear again upon addition of more solvent with vortexing. One intrinsic difference between amorphous dispersion and colloidal complex is the molecular-level mixing of the drug and the polymer. As a necessary step in generating dispersions, it is important to suppress premature precipitation and uncontrolled aggregation. Therefore, during sample preparation, the stepwise addition approach was employed, along with additional solvents and vigorous vortexing to obtain clear solutions.

These solutions containing EGCG and polymer were lyophilized to yield solids. The lyophilized materials are fluffy and lightweight; the Soluplus^®^ dispersion shows a white color while all the others (including the pure EGCG) display some yellowness. Solids were then characterized by XRPD, and the results are also included in Table 1. Among them, Gelucire^®^ 50/13 was not a suitable candidate to generate amorphous dispersion since the lyophilized sample displayed a disordered XRPD pattern with characteristic peaks consistent with Gelucire^®^ 50/13. As a result, four polymers, i.e., HPMCAS, HPMCP, Soluplus^®^, and cellulose acetate, were selected as candidate excipients to generate amorphous dispersions with EGCG for further study.

### 2.2. Characterization of Lyophilized Materials

**Figure 3:**
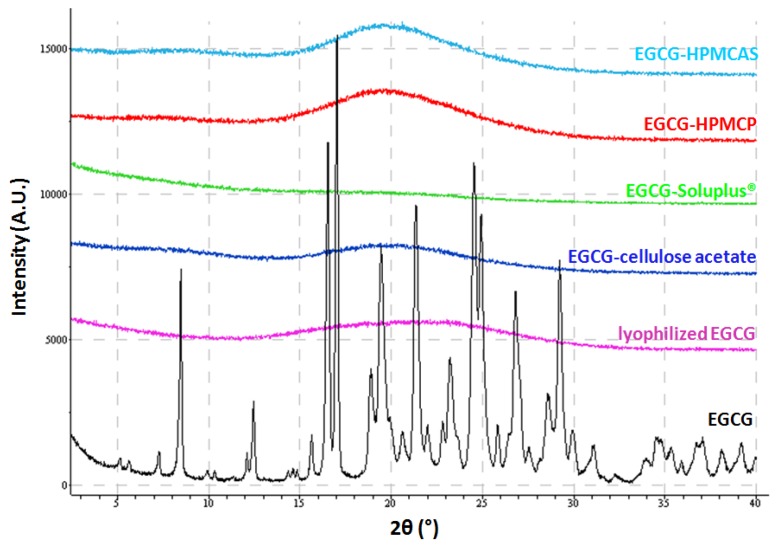
The XRPD patterns of the four dispersions, aEGCG, and cEGCG.

EGCG amorphous solid dispersions generated by lyophilization were characterized by X-ray powder diffraction (XRPD) and data are compared to the lyophilized EGCG and the starting material of EGCG, as shown in Figure 3. Based on the results, the four dispersions and the lyophilized EGCG show clear X-ray amorphous patterns while the EGCG isolated from Teavigo^TM^ is a crystalline material. In later context, the lyophilized EGCG is referred as amorphous EGCG (aEGCG) while the initial EGCG from Teavigo^TM^ is crystalline EGCG (cEGCG).

**Figure 4:**
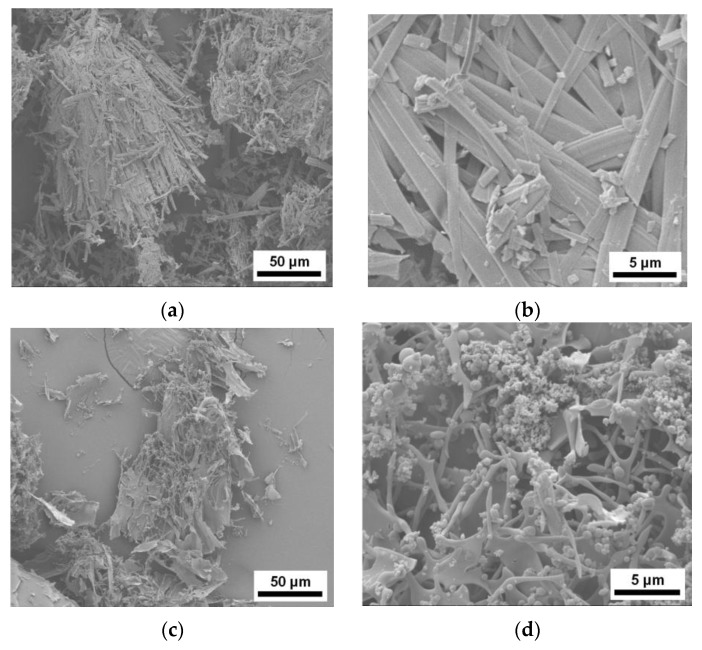

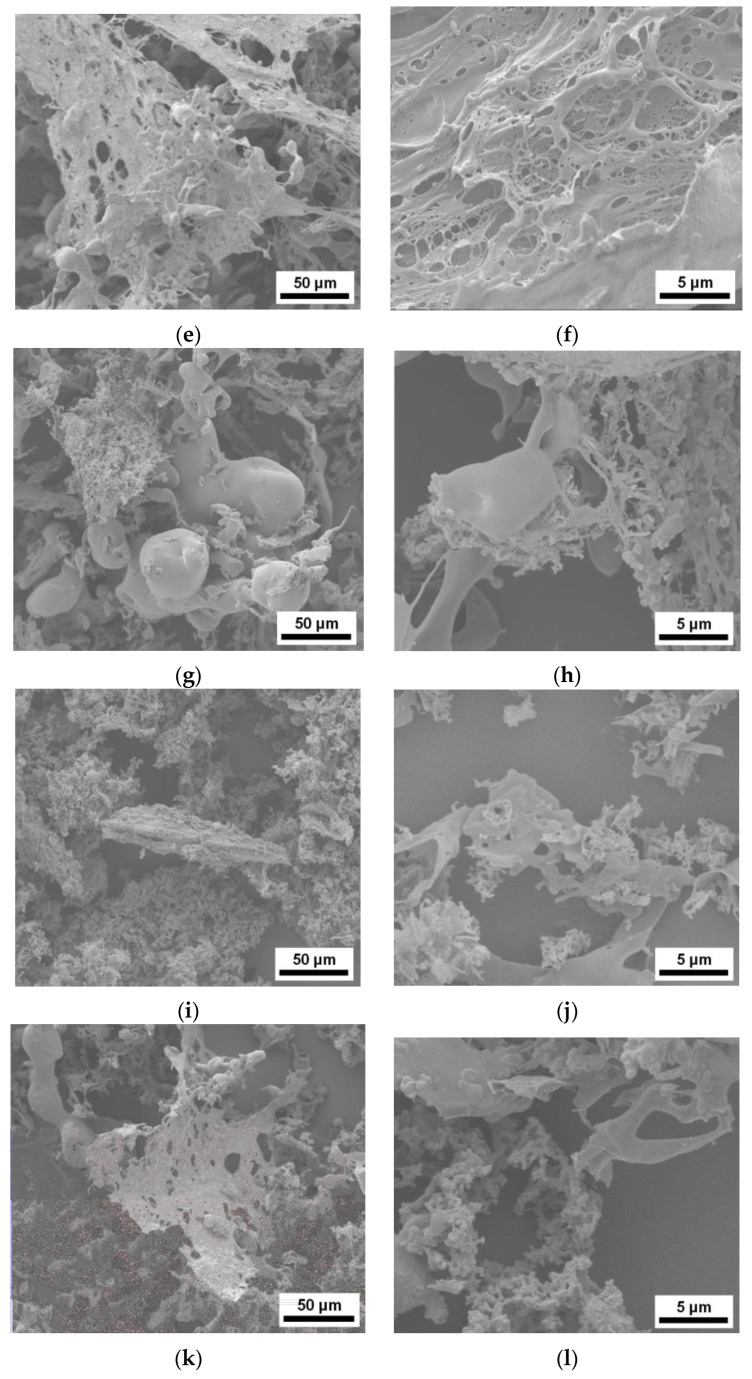
SEM images of (**a**,**b**) cEGCG; (**c**,**d**) aEGCG; and dispersions of EGCG with (**e**,**f**) HPMCAS; (**g**,**h**) HPMCP; (**i**,**j**) Soluplus^®^; and (**k**,**l**) cellulose acetate.

SEM images of cEGCG, aEGCG, and the four dispersions are provided in Figure 4. The cEGCG (Figure 4a,b) and aEGCG (Figure 4c,d) exhibit disparate morphologies. The cEGCG contains long thin flat laths while the aEGCG has continuous structure consisting of round-ended fibers and small spheres with a broad distribution of particle sizes down to tens of nm. The four dispersions all show continuous structures with different morphologies. For the one with HPMCAS (Figure 4e,f), EGCG particles seem intimately interconnected with the polymer forming a porous layered network. The dispersions with HPMCP (Figure 4g,h) and cellulose acetate exhibit relatively separated phases of EGCG and the polymers. The Soluplus^®^ dispersion shows a higher level of homogeneity and no obvious separation is noticed between EGCG and polymer. These morphologies will be further discussed in later context.

**Figure 5:**
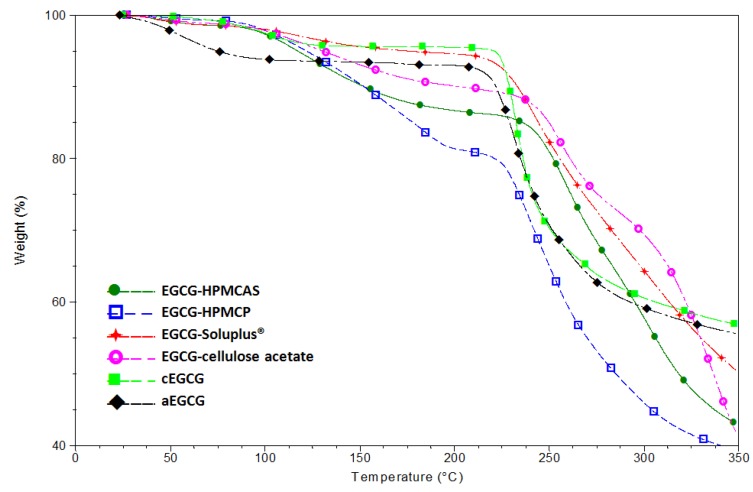
TGA results of the four dispersions, aEGCG and cEGCG.

Figure 5 provides the TGA data of the four dispersions, and both aEGCG and cEGCG. Certain amounts of volatiles are observed in all samples. For EGCGs, water is the volatile while for the dispersions, volatiles could be water, dioxane, or a mixture of both. The amount of volatiles are estimated and accounted for when calculating the EGCG equivalency for dissolution testing. Starting from approximately 220 °C, all materials show apparent decomposition.

**Figure 6:**
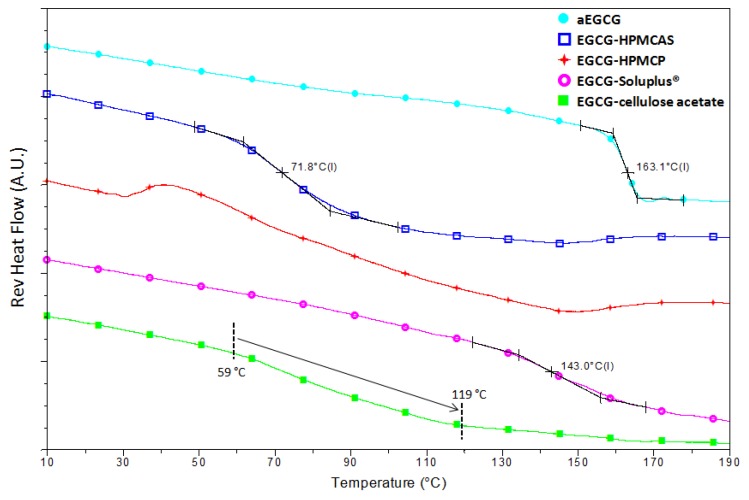
mDSC results of the four dispersions and aEGCG.

Figure 6 shows the mDSC data for aEGCG and the four dispersions. *T_g_*s are about 163 °C, 72 °C, and 143 °C for aEGCG, the dispersions of EGCG-HPMCAS and EGCG-Soluplus^®^ respectively. No clear glass transition is observed for the EGCG-HPMCP dispersion. For the EGCG-cellulose acetate dispersion, the glass transition seems spanning an unusually large range of 59–119 °C. The presence of one (miscible) or two (phase separation) glass transition is typically used to evaluate the drug−polymer miscibility [43]. The EGCG-HPMCAS and EGCG-Soluplus^®^ dispersions display only one apparent *T_g_*, suggesting that EGCG and the polymers are likely miscible in the materials, while the irregular results for EGCG-HPMCP and EGCG-cellulose acetate could be related with phase separation between EGCG and the polymers. These results are consistent with the observations on their microstructural morphologies (Figure 4).

### 2.3. Physical Stability

The physical stability of aEGCG and the four dispersions were evaluated at different conditions including a typical stress condition of 40 °C/75% RH for 11 days, and in media of SGF and SIF for 24 h. Table 2 summarizes the visual observations on the materials after stressing. The aEGCG became a hard material after stress, while all the dispersions were particles or soft aggregates.

**Figure 7:**
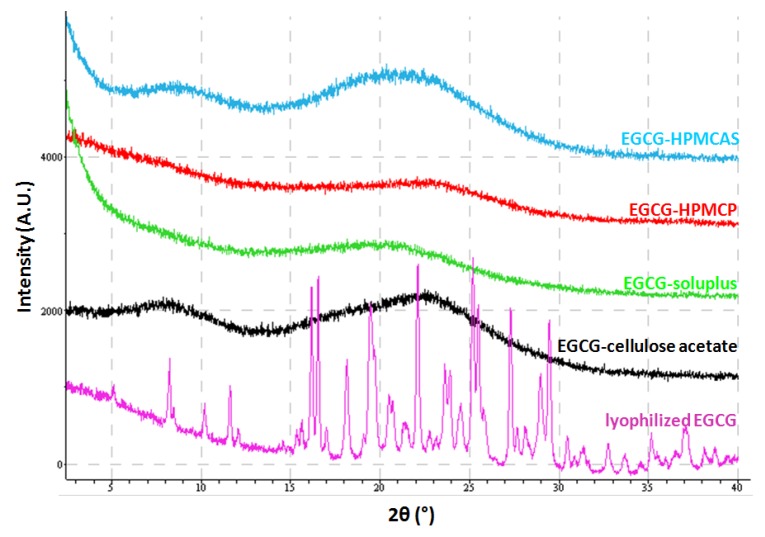
XRPD patterns of the dispersions and aEGCG after stressed at 40 °C/75% RH for 11 days.

The post-stress samples were characterized by XRPD and the results are presented in Figure 7. The aEGCG (lyophilized EGCG) turned into a highly crystalline material while the four dispersions remained X-ray amorphous, suggesting the dispersions are physically stable at this stress condition. In addition, the Soluplus^®^ dispersion remained its white color after stress while all the others presented different levels of color changes. It has been well established that the color change of EGCG is directly correlated with its chemical instability, i.e., degradation and oxidation [26,44]. This difference in coloration after stress may indicate that the Soluplus^®^ dispersion has a higher chemical stability than other dispersions and aEGCG. Further investigation will be needed to confirm and quantify the enhancement in EGCG chemical stability. The improvement in the overall stability will have significant impacts on the whole cycle of storage, transportation, and processing of EGCG.

For the stress testing in media, aEGCG and the dispersions were suspended in SGFs and SIFs, and the suspensions were observed under PLM from time to time to look for evidence of birefringence and extinction (B/E), which is an indication of crystallization of a material. The observations are summarized in Table 3. aEGCG was crystallized quickly, within 15 min in both SGF and SIF; while no occurrence of crystallization was noticed for the four dispersions in both media for at least 24 h, suggesting improved physical stability in the GI environment of the dispersions compared to aEGCG.

### 2.4. Drug Release

**Figure 8:**
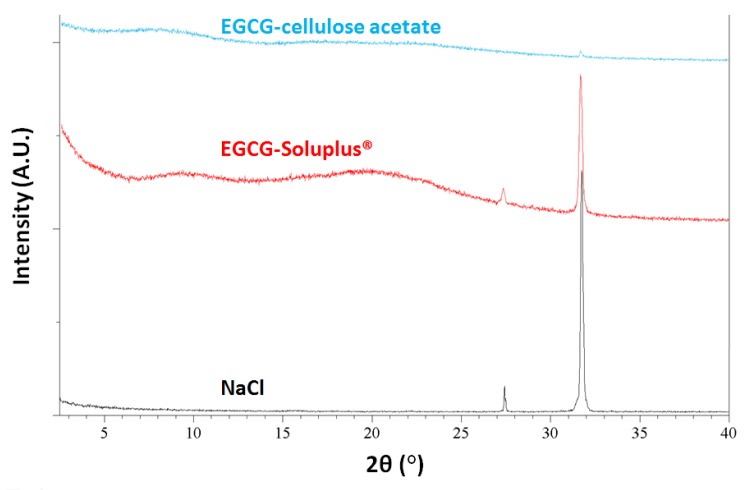
The XRPD patterns of the post-dissolution solids from EGCG-Soluplus^®^ and EGCG-cellulose acetate dispersions.

The dissolution tests for the EGCG and dispersions were carried out in 100 mL standard PBS (pH 7.4) at 37 °C to determine the release profiles of EGCG in the GI tract. For each material, an equivalency of 20 mg EGCG was tested and the experiment was monitored until all solids were dissolved or up to 24 h EGCG concentration in the solution was continuously measured with a UV-Vis dip probe. During the tests, cEGCG, aEGCG, EGCG dispersions prepared with HPMCAS and HPMCP were all dissolved without visible solids, while residual solids were present for dispersions of EGCG-Soluplus^®^ and EGCG-cellulose acetate after 24 h. The solids were isolated, dried under N_2_ purge and then analyzed by XRPD. The XRPD patterns of the post-dissolution solids of EGCG-Soluplus^®^ and EGCG-cellulose acetate dispersions, along with the pattern of NaCl are shown in Figure 8. Clearly, the peaks that appeared in the XRPD patterns of both solids are consistent with NaCl, which came from the dissolution medium. No evidence of crystalline peaks consistent with crystalline EGCG is observed. In fact, based on the dissolution data, for both samples all EGCG (~20 mg) has been released into the medium within the period of dissolution tests. Therefore, the solids isolated after dissolution are un-dissolved polymers, i.e., Soluplus^®^ or cellulose acetate, respectively.

**Figure 9:**
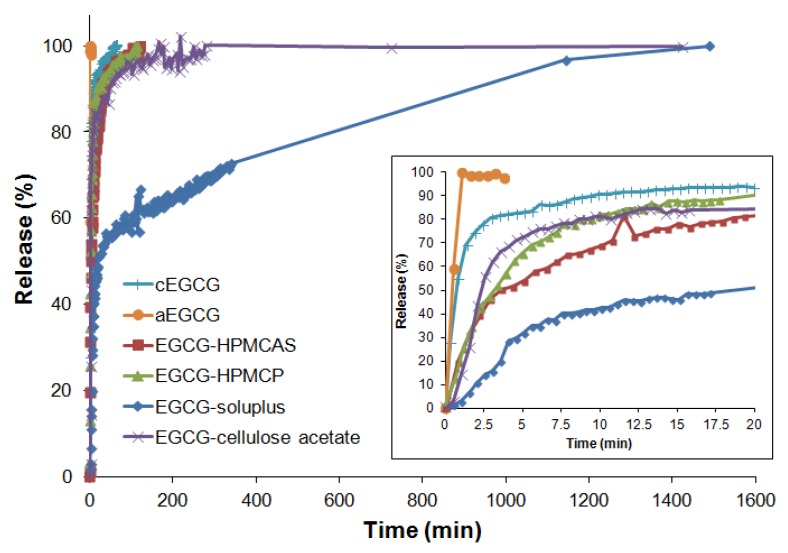
EGCG release profiles for cEGCG, aEGCG, and the dispersions in pH 7.4 PBS medium at 37 °C.

**Figure 10:**
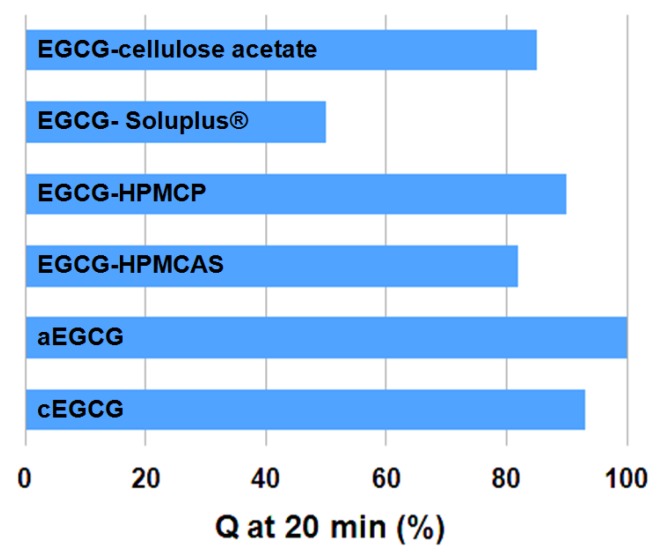
Comparison of EGCG release from the cEGCG, aEGCG, and the dispersions in the first 20 min in pH 7.4 PBS medium at 37 °C.

The release profiles for each material are presented in Figure 9 and the data for the first 20 min are presented in the inset. During the first 20 min, the releases (Q) of EGCG were approximately 93% for cEGCG, 100% for aEGCG, 82% for EGCG-HPMCAS, 90% for EGCG-HPMCP, 50% for EGCG-Soluplus^®^, and 85% for EGCG-cellulose acetate, respectively, as shown in Figure 10.

**Figure 11:**
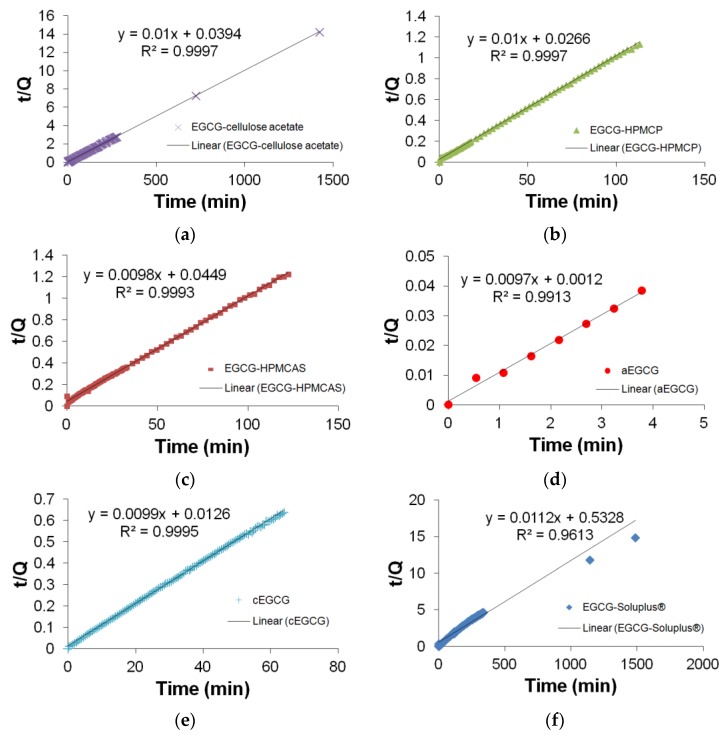
The pseudo second-order fitting for (**a**) cEGCG; (**b**) aEGCG; (**c**) EGCG-HPMCAS; (**d**) EGCG-HPMCP; (**e**) EGCG-cellulose acetate; and (**f**) EGCG-Soluplus^®^.

As expected, aEGCG exhibits an immediate dissolution, at a much higher rate than all the other materials. The three dispersions with HPMCAS, HPMCP, and cellulose acetates show slower release compared to cEGCG. However the extensions are not significant. For dispersions with HPMCAS and HPMCP, since the polymers are quickly dissolved in the medium, they are no longer able to hold EGCG molecules from release. In this case, the interactions between polymers and EGCG do not significantly prolong the release of EGCG in the intestine (high pH). Based on the release profiles for the first 20 min (Figure 9), the dispersions of HPMCP and cellulose acetate have the faster dissolutions than the other two dispersions, which may be due to the apparent separation of EGCG and polymers based on the SEM results presented in Figure 4. A high inhomogeneity is likely to induce an inefficient protection of EGCG from being released into the medium. For the Soluplus^®^ dispersion, a distinct release profile is observed compared to the other materials. Only half of the EGCG is released in the first 20 min and the dissolution continues up to approximately 24 h. To understand the release kinetics, all the profiles are analyzed using the pseudo second-order model:

(1) $$\frac{t}{Q}=\frac{1}{k_{2}Q_{e}^{2}}+\frac{t}{Q_{e}}$$

where t is time, Q the cumulative release, Q_e_ the final release and, and k_2_ the rate constant of pseudo-second order kinetics. The fitting results for t/Q_t_ and t are shown in Figure 11.

Based on the fitting results, releases of EGCG from cEGCG and dispersions with HPMCAS, HPMCP, and cellulose acetate strictly follow the pseudo second-order kinetics model with extraordinarily high correlation coefficients (*R*^2^ = 0.9993–0.9997). aEGCG has a slightly lower *R*^2^ of 0.9935, but can still be considered following pseudo second-order kinetics. The slightly lower *R*^2^ is likely due to the superiorly fast dissolution of aEGCG that there is a delay between the actual release and signal collection by the UV-Vis probe. A distinct nonlinear kinetics is observed for EGCG-Soluplus^®^ dispersion with a significantly low *R*^2^ of 0.9613. It is worth noting that the actual statistical significance may be much smaller than what the *R*^2^ value (0.9613) indicates. From Figure 11, it is clear that after 1000 min the data are quite off the fitting equation while the density of data in this period is also small. It can be expected that if the data density is evenly distributed in the entire testing frame, the *R*^2^ value could be considerably smaller than 0.9613. More importantly, the slope from fitting is 0.0112, which is not reasonable since in pseudo second-order kinetics, this value should not be beyond 0.01 (the reciprocal of 100, final release percentage). This result indicates that pseudo second-order kinetics model is not suitable to explain the release mechanism for EGCG-Soluplus^®^ dispersion. A likely scenario is that there exist two mechanisms: (1) Although EGCG molecules are bound/interconnected with Soluplus^®^ matrix, some EGCG molecules are not safely hidden in the polymer envelops, e.g., they may be on the surface of the polymers or not 360° wrapped. This part of EGCG follows a release mechanism similar to the other dispersions. (2) The rest of the EGCG molecules are tightly surrounded by Soluplus^®^, and can only be released in a much slower rate, i.e., via a second mechanism.

It is well known that Soluplus^®^, as a polyvinyl caprolactam–polyvinyl acetate–polyethylene glycol graft copolymer, has an amphiphilic structure with a high lipophilicity, and possesses a very low critical micelle concentration (7.6 mg/L) [45]. With a bifunctional character, Soluplus^®^ can be used as a matrix polymer for solid solutions and also an active solubilizer through micelle formation in water [46]. A number of reports have demonstrated the use of self-assembled polymer micelles based on amphiphilic block copolymers as vehicles to improve delivery and bioavailability [47,48,49,50,51]. In this study, during the dispersion generation, Soluplus^®^ reached a concentration of 7353 mg/L in water-dioxane solution prior to lyophilization. This concentration is much higher than its critical micelle concentration, and therefore Soluplus^®^ micelles are likely formed in the solution with EGCG molecules inside the micelles. The formation of micelles results in a tight wrapping of the EGCG molecules and/or the excellent miscibility between Soluplus^®^ and EGCG, and therefore the lyophilized solids display a white color while the other dispersions show yellowness originating from the color of EGCG (Table 1). In the dissolution test, the tightly wrapped EGCG can only be released after the dissolution or at least partial dissolution of Soluplus^®^, which could form micelles again in the dissolution medium and further delay the release of EGCG.

With the strong tendency to form micelles, the interaction between EGCG and water is mitigated, which is similar with some previous results [52]. Nagy et al. has succeeded in controlling the release of spironolactone via electrospinning and extrusion using Soluplus^®^ as a drug carrier matrix [53]. On one hand the abundant hydroxyls of EGCG render an adequate binding with the hydrophilic chains in Soluplus^®^. On the other hand the high lipophilicity of Soluplus^®^ appreciably lowers the wettability of EGCG. The inhibition of EGCG-water interaction leads to the second release mechanism, which is inherently different from the first one that EGCG is readily to be dissolved into the medium without the protection of micelles.

As a result of the two distinct mechanisms, a biphasic model that combines pseudo-second-order and first-order kinetics (PSO-FO) is used to describe the release profile of the Soluplus^®^-EGCG dispersion:

(2) $$Q_{t}=\frac{t}{1/k_{2}Q_{e}^{2}+1/Q_{e}}+Q_{0}\left(1-\exp\left(-k_{1}t\right)\right)$$

**Figure 12:**
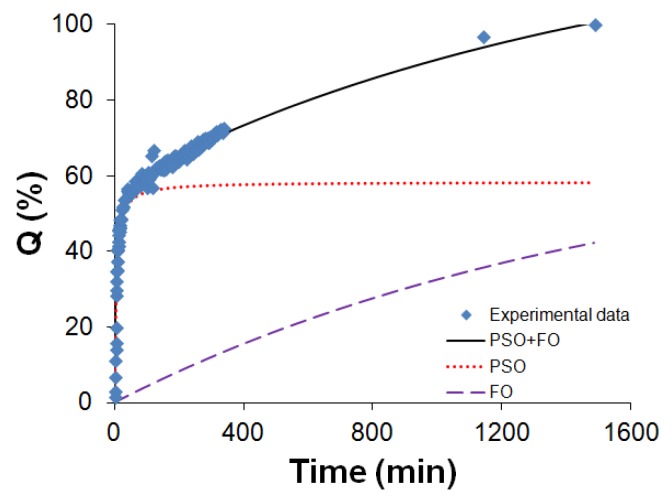
The release profile of Soluplus^®^-EGCG dispersion fitted with biphasic PSO-FO model.

It is noteworthy that the first order model is also a special case of the Weibull model:

(3) $$Q=Q_{0}\left(1-\exp\left(-\frac{{\left(t-T\right)}^{b}}{a}\right)\right)$$

which has been feasible to describe release profiles of matrix type drug delivery [54,55]. The release profile fitting is presented in Figure 12.

The data were fitted by this biphasic PSO-FO model with a high *R*^2^ of 0.9891. The two terms in Equation (2) can be used to estimate the cumulative releases by the two fractions of EGCG– on the polymer surface/open structures (PSO release) and tightly protected EGCG (FO release). For example, at the time of 20 min, the PSO contribution is 47% and FO is 1%; at the time of 24 h, the PSO release is 58% and the FO release is 42%.

Soluplus^®^ has shown superior solubilizing properties for BCS (Biopharmaceutics Classification System) class II drugs and offers the possibility of producing solid solutions of several poorly water soluble drugs [46,56]. In this study, EGCG is a BCS class III drug with good aqueous solubility but poor bioavailability. To the best of the authors’ knowledge, this is the first report using Soluplus^®^ generating a sustained release for water-soluble drug. In addition, Soluplus^®^ micelles have been demonstrated to improve oral bioavailability by altering the membrane permeability in the intestines [57,58]. The EGCG-Soluplus^®^ dispersion generated in this work not only improves the EGCG’s bioavailability by sustained release, but also possibly opens up additional opportunities for bioavailability enhancement by increasing its membrane permeability, which however, requires further in vivo work to confirm. In addition, it is expected that by altering the EGCG/Soluplus^®^ ratio, the drug release profile of EGCG can be conveniently further controlled.

## 3. Experimental Section

### 3.1. Materials

Teavigo^TM^ was purchased from Healthy Origins (Pittsburgh, PA, USA) and EGCG was isolated from it and used as the starting material [33,59]. NF Grade hydroxypropyl methylcellulose acetate succinate (HPMCAS) and hydroxypropyl methylcellulose phthalate (HPMCP) were received from Shin-Etsu Chemical (Osaka, Japan); Soluplus^®^ was from BASF (Florham Park, NJ, USA); cellulose acetate, polyvinylpyrrolidone (PVP K-90), polyvinyl alcohol (PVA), polyvinyl acetate (PVAc), polyethylene glycol (PEG), dioxane, and water (HPLC grade) were purchased from Sigma-Aldrich (St. Louis, MO, USA); Gelucire^®^ 50/13 was received from Gattefosse (Paramus, NJ, USA); simulated gastric fluid (SGF) and simulated intestinal fluid (SIF) were purchased from RICCA Chemical Company (Arlington, TX, USA).

The Teavigo^TM^ solids were suspended in water and filtered to remove the solids. The resultant solution was clear with no evidence of solid particles observed. The solution was then dried under N_2_ purge until water was fully removed based on visual observation. The generated EGCG solids were directly used for preparing solid dispersions with polymers at a composition of 50:50 (*w*/*w*).

EGCG solid dispersions were prepared by lyophilization with five polymers including HPMCAS, HPMCP, Soluplus^®^, cellulose acetate, and Gelucire^®^ 50/13. These polymers were selected based on initial screen results. For the generation of dispersions, individual solutions of polymer (in dioxane) and EGCG (in water) were prepared. The EGCG aqueous solution was added into the polymer-dioxane solution slowly and stepwisely, with each step of about 1–2 mL. For each step, additional fresh dioxane or water may be added into the mixtures followed by vigorous vortexing to reach a clear solution without visible solids or colloids. The approximate ratios of water to dioxane for the final solution of the five dispersions are provided in Table 4.

For lyophilization, each EGCG-polymer solution was frozen in a cold bath of dry ice/acetone (at a temperature of −78 °C). The sample vial was then attached to a Flexi-Dry manifold lyophilizer (SP Industries, Stone Ridge, NY, USA) at −50 °C for 3 days. Pure EGCG aqueous solution was lyophilized as well to generate amorphous EGCG (aEGCG) as a control for comparison with the dispersions. After lyophilization, the dispersions were further dried under vacuum at 40 °C for 5 days, and the aEGCG was dried under vacuum at room temperature (RT) for 8 days, to remove the residual solvents (dioxane and water).

### 3.2. Analytical Instruments for Characterization

The X-ray powder diffraction (XRPD) data were collected with a PANalytical X’Pert PRO MPD diffractometer. Polarized light microscopy (PLM) was performed using a Leica DM LP microscope with a cross polarizer. The thermogravimetric analyses (TGA) were performed using a TA Instruments Q5000 IR thermogravimetric analyzer. Each sample was placed in an aluminum sample pan and inserted into the TGA furnace. The furnace was first equilibrated at 25 °C, and then heated under N_2_ at a rate of 10 °C/min, up to a final temperature of 350 °C. Modulated differential scanning calorimetry (mDSC) data were obtained on a TA Instruments Q2920 differential scanning calorimeter equipped with a refrigerated cooling system. The test was progressed by modulating temperature ±0.8 °C every 60 s from 0 to 180 °C. The reported glass transition temperature (*T_g_*) is obtained from the inflection point of the step change in the reversing heat flow versus temperature curve.

### 3.3. Stress

The physical stability of the dispersions is evaluated under different stress conditions including exposure to elevated temperature/relative humidity (RH) and in aqueous media. In the elevated temperature/RH condition, samples were exposed to approximately 40 °C/75% RH for 11 days, and then characterized by XRPD for evidence of crystallization. The samples were also suspended in simulated gastric fluid (SGF) and simulated intestinal fluid (SIF), and observed under PLM from time to time up to 24 h for evidence of crystallization based on the appearance of birefringence/extinctions. These stress conditions simulate two different end influences on EGCG: at storage/processing and in the GI tract.

### 3.4. Dissolution

Dissolution testing was performed in 100 mL standard phosphate-buffered saline (PBS, pH 7.4) at 37 °C in a jacketed beaker. The temperature was precisely controlled by a Julabo Heating Circulator. EGCG concentration in the solution was in situ monitored via an Ocean Optics USB2000+ UV-VIS spectrometer (Ocean Optics Inc., Wesley Chapel, FL, USA) equipped with an RT-2MM Dip Probe. The testing was performed with continuous stirring until all solids were dissolved and the medium was free of observable particles, or up to 24 h. For early period of dissolution, the data acquisition density is relatively large to capture all sudden changes; while at later periods, the data acquisition frequency was reduced due to slow evolution in the medium.

## 4. Conclusions

Four amorphous solid dispersions of EGCG are successfully generated targeting two significant issues of this promising tea extract: high physical instability and low bioavailability. The physical stability is found to be greatly enhanced under two typical stress conditions, 40 °C/75% RH and pH conditions that simulate the GI tract. These improvements are encouraging with respect to the invulnerability during storage/processing and the diminished degradability in dosing. In addition, the EGCG-Soluplus^®^ dispersion shows a significantly sustained release behavior compared to pure EGCG, which may potentially improve the oral bioavailability, considering the rapid in vivo elimination of EGCG. This solid dispersion approach is an easy method that does not involve strict experimental conditions, which may offer a big step forward in the marketing and efficacy of this green tea extract with great potential.

## Figures and Tables

**Table 1:** Initial screen results of nine polymers with EGCG.

Polymer	Visual Observation upon Mixing	Visual Observation after Lyophilization	XRPD Results
HPMCAS	clear solution	light yellow fluffy particles	X-ray amorphous
HPMCP	clear solution	yellow fluffy particles	X-ray amorphous
Soluplus^®^	clear solution	white fluffy particles	X-ray amorphous
Cellulose acetate	clear solution	light yellow fluffy particles	X-ray amorphous
Gelucire^®^ 50/13	clear solution	light yellow fluffy particles	disordered
PVA	flocculated suspension	-	-
PVP	flocculated suspension	-	-
PEG	flocculated suspension	-	-
PVAc	flocculated suspension	-	-

**Table 2:** Observation of the materials after 40 °C/75% RH stress for 11 days.

Material	Visual Observation after Stress
aEGCG	pink hard material, agg.
EGCG-HPMCAS	pink agg. and particles
EGCG-HPMCP	pink agg. and particles
EGCG-Soluplus^®^	white free flowing agg. and particles
EGCG-cellulose acetate	dark pink agg. and particles

**Table 3:** Observations under PLM for the dispersions and aEGCG in SGF and SIF.

Material	in SGF	in SIF
aEGCG	B/E observed in 15 min	
EGCG-HPMCAS	no B/E observed in 24 h	
EGCG-HPMCP		
EGCG-Soluplus^®^		
EGCG-cellulose acetate		

**Table 4:** Final mixtures proportions of the four dispersions.

	EGCG (g)	Polymer (g)	H_2_O (mL)	Dioxane (mL)
HPMCAS	1	1	50	80
HPMCP	1	1	58	100
Soluplus^®^	1	1	58	78
Cellulose acetate	1	1	58	140
Gelucire^®^ 50/13	1	1	58	325
